# A commissioning procedure for breast intracavitary electronic brachytherapy systems

**DOI:** 10.1120/jacmp.v9i3.2775

**Published:** 2008-06-23

**Authors:** Jessica Hiatt, Gene Cardarelli, Jaroslaw Hepel, David Wazer, Edward Sternick

**Affiliations:** ^1^ Department of Radiation Oncology Rhode Island Hospital Providence RI U.S.A.

**Keywords:** Electronic Brachytherapy, commissioning, Intracavitary Accelerated Partial Breast Irradiation

## Abstract

In this work, we report a comprehensive quality assurance (QA) process for the commissioning of an Electronic Brachytherapy (EB) system at one of the first U.S. sites to apply the device clinically. Thus far, EB systems have been released only for intracavitary breast treatments. As such, EB as an Accelerated Partial Breast Irradiation (APBI) treatment modality is relatively unstudied and is unfamiliar to many medical physicists. We present our documented experience as a guide for other institutions' EB commissioning process. Our tests included eight elements: A) well‐chamber constancy, B) beam stability, C) source positional accuracy, D) output stability, E) timer linearity, F) dummy marker/source position coincidence, G) controller functionality and safety interlocks, and H) treatment planning data verification following the AAPM TG‐43 recommendations. Together with TG‐43, our methodology provides a comprehensive EB system check for medical physicists commissioning such a device.

PACS Numbers: 87.53.Jw, 87.53.Xd

## I. INTRODUCTION

For patients with early‐stage breast cancer, breast conservation therapy (BCT) has become the standard of care. Traditional BCT consists of a lumpectomy followed by a daily treatment course of whole breast external beam irradiation for approximately six weeks. A number of clinical studies ^1^, ^4^ have shown that most ipsilateral breast recurrences after BCT arise in the peripheral lumpectomy cavity, implying that whole breast irradiation might be unnecessary for these patients. Consequently, the practice of treating only a limited volume of breast tissue surrounding the lumpectomy cavity (1 to 2 cm for patients with negative margins) has been introduced. This type of treatment is termed Accelerated Partial Breast Irradiation (APBI). Because the treated volume is relatively small with APBI, the dose per fraction delivered can be increased significantly, and the overall treatment time reduced to one week.

One of the most widely used APBI techniques, intracavitary partial breast irradiation, employs a saline‐filled balloon with an attached after‐loading catheter inserted within the lumpectomy cavity.^5^, ^9^ Depending on lumpectomy cavity geometry, this balloon can be either spherical or ellipsoidal, with diameters ranging from 4–5 cm or 6–7 cm. A high dose rate (HDR) Ir‐192 source is inserted through the catheter and delivers the treatment following balloon placement.

A new APBI approach, electronic brachytherapy (EB), is provided by the Axxent Electronic Brachytherapy System (Xoft Inc., Fremont, CA). Similar in concept to the Ir‐192 intracavitary system, the Xoft Axxent System also uses a balloon catheter surgically implanted in the excision cavity. Unlike HDR delivery with a radioactive source, however, the Axxent System instead controls the travel of a micro‐miniature, water‐cooled 50 kV X‐ray source through the balloon catheter. The source is turned on at appropriate positions within the balloon at preprogrammed dwell times to deliver the prescribed dose.

The EB modality has several advantages. Because of the relatively low energy of the Xoft device, treatments can be delivered in an unshielded room in contrast to the significant bunker shielding required for HDR brachytherapy. The low exposure rate also allows staff to remain near the treatment couch during dose delivery, offering an opportunity to provide comfort and encouragement while in close proximity to the patient.

Our institution was one of the first U.S. sites to treat patients with the Axxent System for EB. The objective of this paper is to describe the physics commissioning procedures that we designed and implemented prior to its clinical use.

## II. MATERIALS AND METHODS

The report of the American Association of Physicists in Medicine (AAPM) Task Group 43^10^, ^11^ offered recommendations for HDR brachytherapy quality assurance and dose calculations and provided guidance for the tests we designed for commissioning the Xoft Axxent System. Source design, dosimetric properties, such as dose distribution and isotropic nature, and recommendations for clinical implementation for posting on the joint AAPM/RPC Brachytherapy Source Registry have been published by Rivard et al.^(12)^

**Figure 1:**
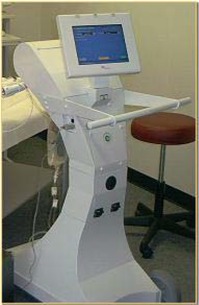
Xoft Axxent Controller.

**Figure 2:**
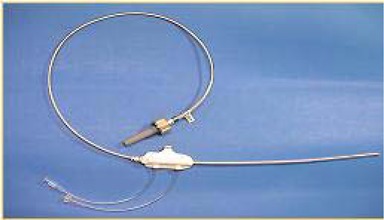
Xoft Axxent 50 kV Source.

**Figure 3:**
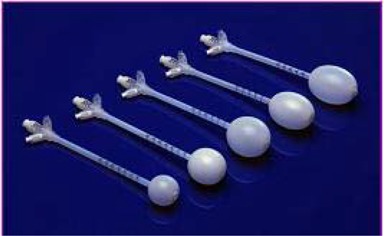
Intracavitary breast balloon applicators.

System components consist of the Xoft Controller (Fig. 1), which includes a touch‐screen monitor, USB port, pull‐back arm (adjustable arm with a high voltage port for the source connection), bar‐code scanner, X‐ray source cooling pump, Standard Imaging well chamber, and a Standard Imaging Max‐4000 electrometer; Xoft Axxent 50 kV sources (Fig. 2); and the applicator balloons ranging in spherical size from 3 – 4 cm to 5 – 6 cm diameter and in ellipsoid size from 6×7 cm to 5×7 cm (Fig. 3). For exposure to air kerma strength conversions, the University of Wisconsin (UW), Department of Medical Physics, Accredited Dosimetry Calibration Laboratory, has provided a calibration coefficient for the Axxent source. This procedure is described in the UW application note 06–01 which uses the laboratory's standard HDR 1000 well chamber and compares the Xoft Axxent 50 KV source to Amersham Model 6711 I‐125 seed source.

The commissioning tests included: A) Well‐chamber constancy, B) Beam stability, C) Source positional accuracy, D) Output stability, E) Timer linearity, F) Marker/source position coincidence, G) Controller functionality and safety interlocks and H) Treatment planning data verification of TG‐43 parameters.

### A. Well‐chamber constancy

The first step in commissioning the EB system was to intercompare our department's well chamber/electrometer and the well chamber/electrometer integrated into the Axxent treatment console to verify the proper functioning of the manufacturer‐provided measurement system. Both systems consist of a Standard Imaging HDR 1000 Plus well chamber and a Standard Imaging Max 4000 electrometer. The intercomparison test was performed using a 4 mg Radium‐equivalent (Ra‐eq) Cesium‐137 source and a fabricated source calibration jig that allowed for reproducible placement of the source within the well chamber.

### B. Beam stability

Data from a simulated treatment was extracted from the controller unit and analyzed to determine the stability of the beam throughout a treatment fraction.

### C. Source positional accuracy

A test of the coincidence between the planned and actual source positions was performed as follows.

First, a “dwell file” was created in a Microsoft Excel spreadsheet using the Xoft‐provided software add‐in. The dwell file defines the source position and dwell time information for each plan. It is created in Excel and then transferred to the treatment console via USB flash drive.

**Figure 4:**
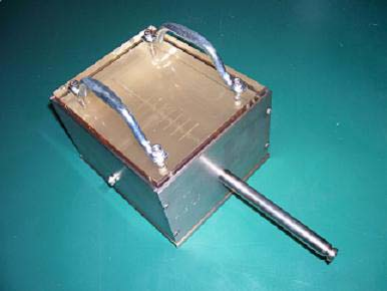
QA test fixture consisting of a shielded box with leaded glass slabs and inserts for the source, film and ion chambers.

The dwell file contained data directing the source to stop for 10 second intervals at distances specified to be 24.5, 23.5, 22.5, 21.5 and 20.5 cm. The dwell file data were imported into the treatment console, the source calibrated and the system connected to the QA test fixture provided by the manufacturer (Fig. 4).

The QA test fixture consists of two blocks of acrylic in lead housing. The lower slab contains a channel fitted for the X‐ray source with a second, perpendicular channel 1 cm below the source channel for the placement of either a Farmer or pinpoint chamber. The top portion of the lower slab includes thin horizontal lead graduations. The graduations are separated by 1 cm lengths to correspond to regularly spaced dwell positions. A sheet of GafChromic film was placed 1 cm above the source and beneath the second acrylic slab. By positioning the film in this arrangement, the beam will be attenuated at the programmed dwell positions by the lead graduations and a visual inspection of planned versus actual dwell position can be performed.

A second test to assess the expected/actual source position coincidence was also performed. A dwell file was created consisting of 15, 10 second dwell positions separated by 0.5 cm. With the X‐ray source located at the first dwell position, the distance from the source hub to the proximal end of the QA test fixture was recorded for each dwell position using digital calipers.

### D. Output stability

The next test performed was a beam output stability test. Relative rate (nA) measurements were obtained using a PTW Pinpoint chamber (Model 31006, SN0299) set 1 cm from the source within the QA test fixture. The instantaneous current was measured when the source had arrived at each of its programmed positions. The electrometer leakage was measured before the data collection to be less than $0.005\times 10^{‐11}\;A$.

### E. Timer linearity

The linear response of the charge collected as a function of time was determined by recording the collected charge for set dwell times. Four dwell files were created, with times varying from 30 to 120 seconds at a dwell position of 22 cm. This dwell position placed the source directly above the pinpoint chamber within the test fixture.

### F. Marker/source position coincidence

To ensure that the dummy marker and source positions were coincident within the applicator so as to provide an accurate treatment plan when defining the first dwell position, we stapled XV film to a GafChromic film and affixed a Xoft balloon applicator. A three‐dwell position file was specified. With the dummy marker in place, a large field on the film was exposed using the department's conventional X‐ray unit. The treatment was then administered to the balloon/film arrangement. Low exposure XV (fast speed) film was required to visualize the dummy markers after conventional X‐ray exposure; high exposure GafChromic (slow speed) film was required to visualize the source positions after exposure using the Xoft Axxent 50 kV source. The films were processed, scanned and analyzed.

### G. Controller functionality and safety interlocks

A number of tests were performed to evaluate overall system performance and the safety of the device.

### H. Treatment planning data verification of TG‐43 parameters

The manufacturer provided TG‐43 physics data as published by Rivard et al.^(12)^ for input into the PLATO (Nucletron Corporation, Columbia, MD) treatment planning software (TPS). These data included: $S_{k}$ – air kerma strength, Λ‐ dose rate constant, $g_{P}(r)$ – radial dose function, $G_{P}(r,\;\theta )$ – line source geometry function and F(r, θ) – anisotropy function. Data is also available for Varian's Brachyvision (Varian Medical Systems, Palo Alto, CA) treatment planning program as the Xoft Axxent system does not come with its own treatment planning system. During the treatment planning phase, a nominal source strength is used. Before treatment the dwell times are adjusted to account for the source strength at that time; in this way, there are no source decay issues in the TPS. An in‐house HDR second check program was used to verify the dose calculated by our PLATO TPS. The second check program uses the TG‐43 parameters of the source, requiring the input of dwell times, dwell position coordinates, dose point coordinates and the planned dose at those points and outputs the independently determined dose for comparison using a 3D inverse square vector analysis.

**Figure 5:**
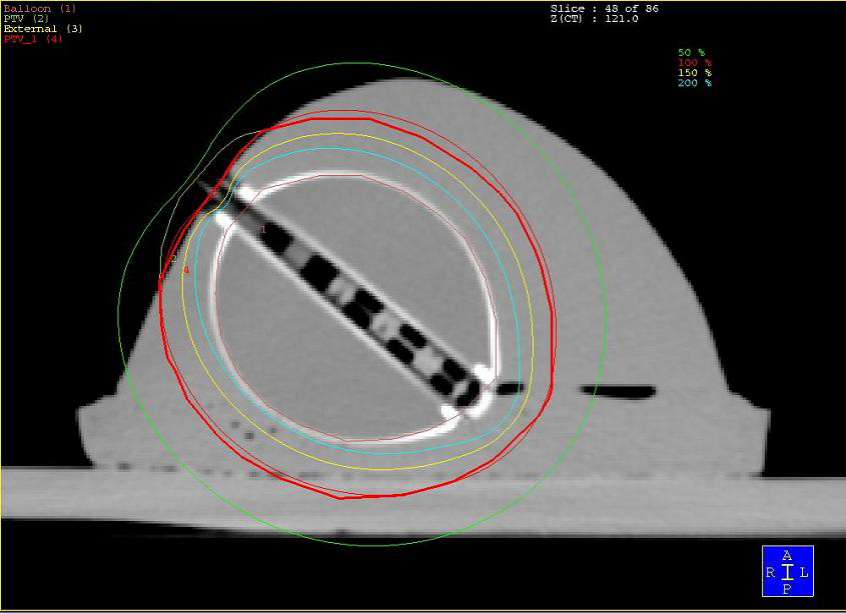
A gelatin breast phantom with the balloon applicator implanted. This axial CT treatment‐planning image depicts the 50, 100, 150 and 200% isodose lines.

To compare the planned‐to‐actual dose, a gelatin breast phantom was fashioned with the Xoft balloon applicator embedded to simulate realistic patient geometry. The phantom was scanned by CT, the images sent via DICOM transport to the treatment planning system and a plan created using standard intracavity accelerated partial breast fractionation schema and target delineation.^(13)^ An axial CT treatment planning image of the phantom is shown in Fig. 5. Landauer DOT dosimeters were placed on the phantom at specified points where the balloon surface to skin distance was the least. DOT dosimeters utilize optically stimulated luminescence technology to provide radiation monitoring and are designed for dose measurements in the kilo‐voltage range.^(14)^ Results of the breast phantom treatment planning study are pending publication.

## III. RESULTS AND DISCUSSION

### A. Well‐chamber constancy

Three trials were performed with each well‐chamber/electrometer measurement system; the results are listed in Table 1. Both systems passed the constancy check, showing differences of less than 2%. The difference between the two systems was 0.14%.

**Table 1:** Data from the well‐chamber and electrometer intercomparison using a 4 mg Ra‐eq Cs‐137 source for the in‐house and the Xoft controller measurement systems.

	*Well‐Chamber Constancy Check*			*% Difference*
RIH^*^ Unit Readings (pA)	41.928	41.892	41.885	−1.89
Xoft Unit Readings (pA)	41.944	41.967	41.976	−0.73

* RIH=Rhode Island Hospital

### B. Beam stability

**Figure 6:**
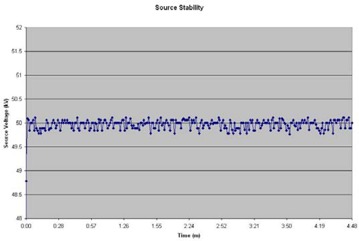
Source voltage plotted as a function of time (5 minute interval).

Beam stability was assessed by analyzing the log‐file for a given test run. The log‐file contains the calibration data from the electrometer, the calibration factor corrected for temperature and pressure, dwell times from the treatment planning system, scaled dwell times after source calibration and lists any errors that may have occurred during treatment. The generator voltage and current and beam current were plotted versus time and studied for drift from the set‐point (Fig. 6). The system was found to have excellent beam constancy.

### C. Source positional accuracy

**Figure 7:**
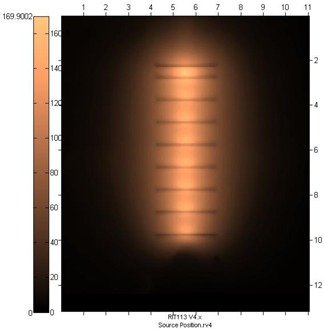
RIT GafChromic film image displaying agreement between actual and planned source positions. The light colored areas represent the dwell positions; the horizontal lines represent the planned positions. Due to the inherent forward anisotropy, the horizontal line appears slightly below the apparent center of the dose cloud.

Following exposure, the film was scanned using RIT software (Radiological Imaging Technology, Colorado Springs, CO) for analysis of source planned and actual position coincidence (Fig. 7). The source was found to overlay the marking denoting each planned dwell position.

For the second source positional accuracy test, each measurement was recorded in Table 2 and the measured step lengths determined to assess pullback accuracy. The total measured pullback distance was determined to be 69.88 mm versus a planned pullback distance of 70.00 mm. The 0.02 mm discrepancy yielded a difference of 0.2%.

**Table 2:** Planned dwell position, step length, caliper reading and measured step length for a given dwell file. Pullback Accuracy Test

*Dwell Number*	*Dwell Position (cm)*	*Step Length (mm)*	*Caliper Reading (mm)*	*Measured Step Length* $\left(mm)(d_{n}-d_{n-1}\right)$
1	24.5	5.0	16.45	
2	24.0	5.0	21.42	4.97
3	23.5	5.0	26.81	5.39
4	23.0	5.0	32.03	5.22
5	22.5	5.0	36.34	4.34
6	22.0	5.0	41.20	4.86
7	21.5	5.0	46.64	5.44
8	21.0	5.0	51.38	4.76
9	20.5	5.0	56.27	4.89
10	20.0	5.0	61.42	5.15
11	19.5	5.0	66.32	4.90
12	19.0	5.0	71.61	5.29
13	18.5	5.0	76.42	4.10
14	18.0	5.0	81.28	4.86
15	17.5	5.0	86.33	5.05
	$d_{15}$ – $d_{1}$	**70.0**		**69.88**

### D. Output stability

Five trials observing the output stability at each of the dwell positions were performed. The data are reported in Table 3. With a relative standard deviation of less than 0.02 nA among trials, the beam output was found to be stable.

**Table 3:** Instantaneous rate readings for a five‐dwell position plan.

	*Trial Number*							
*Rate (nA) Dwell Position (cm)*	*1*	*2*	*3*	*4*	*5*	*Average*	*Standard Deviation*	*Relative SD*
24.5	0.006	0.006	0.006	0.006	0.006	0.006	0.000	0.00
23.5	0.022	0.022	0.022	0.022	0.022	0.022	0.000	0.00
22.5	0.110	0.112	0.111	0.111	0.111	0.111	0.001	0.01
21.5	0.190	0.199	0.199	0.200	0.199	0.197	0.004	0.02
20.5	0.077	0.079	0.080	0.080	0.080	0.079	0.001	0.02
Total Rate (nA)	0.405	0.418	0.418	0.418	0.418	0.416	0.006	0.01

### E. Timer linearity

**Figure 8:**
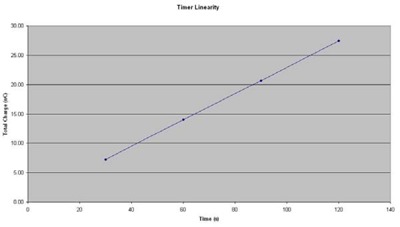
Source linearity.

The graphical results of charge versus time are shown in Fig. 8. Three consecutive charge readings were recorded for each of the planned times. Collected charge and dwell time were found to be perfectly correlated (Table 4), with a correlation coefficient of 1.00. The timer error was estimated to be 2.83 seconds by extrapolation. The error would be considered significant for dwell times less than 28 seconds.

**Table 4:** Linear increase of collected charge with increasing beam‐on time.

*Timer Accuracy and Linearity*						
Time (sec)	120	90	60	30	Timer Error (sec)	2.83
Averace Reading (nC)	27.62	20.92	14.18	7.36	Correlation	1.000

### F. Marker/source position coincidence

**Figure 9:**
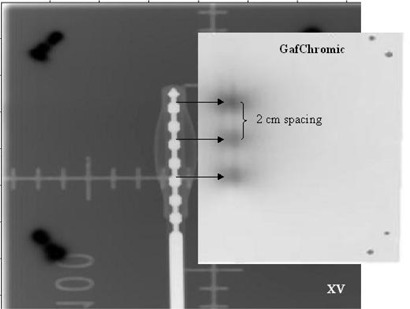
A composite image created in RIT representing the marker catheter (XV film, left) and actual source positions (GafChromic film, right). The films were aligned by the shared staple positions and then offset to visualize the coincidence between planned and actual dwells. The dwell file consisted of 3 positions with 2 cm spacing.

In the film scanning software, the staple positions were used to register and superimpose the films. In this way, a composite image was created showing both the dummy and source positions (Fig. 9). By visual inspection and measurement, it was determined that the coincidence was satisfactory.

### G. Controller functionality and safety interlocks

The final system tests consisted of an overall controller functionality evaluation:

- The setup and delivery were tested by entering a test patient's data manually into the system using the touch screen monitor. A dwell file was imported into the system via USB flash drive. The source was calibrated using the well‐chamber/electrometer combination. Ambient temperature and pressure readings were entered into the system to provide a correction factor for the source calibration ratio. The treatment was then delivered to the QA test fixture. Post‐treatment, a log‐file was copied to the USB flash drive. This file was reviewed for accuracy and source stability.
- The status indicator light performance was verified during the treatment. The status indicator light is required to alert users that radiation is currently being emitted.
- The emergency‐off button was tested to ensure that when pressed, the source would stop emitting radiation and the system would turn itself off.
- The treatment recovery procedure was tested to verify that in the case of an emergency‐off or a power failure, the treatment could be restarted and continue to completion following recovery.
- A sampling of applicators was tested for symmetry when filled with saline.
- The source was observed during travel through a bent applicator to ensure that the controller would register a pullback force error with the catheter in this incorrect configuration and would not go into treatment mode.

### H. Treatment planning data verification of TG‐43 parameters

The treatment plan created in PLATO was verified using an in‐house independent second check program. The maximum dose difference between the treatment planning data and the second check program was 1.2%.

Using the breast phantom described above, we compared the planned dose to a specified point to the actual dose using dosimeters rated for dose readings in the kV range. Measurements were made on three dosimeters placed at each of the seven specified points. The greatest deviation between planned and measured dose to the selected points was found to be 3.18%.

## V. CONCLUSION

Only recently introduced as a commercially available system, EB has been relatively unstudied thus far in the clinical setting and is consequently unfamiliar to many medical physicists. To date, there have been no publications that document the proper commissioning process. The methods described in this paper chronicle our Xoft Axxent Electronic Brachytherapy system commissioning experience and the obtained results. Along with AAPM TG‐43, these procedures can be used as an acceptance and commissioning protocol of EB devices for intracavity breast treatments.
